# Hepatic Cellular Distribution of Silica Nanoparticles by Surface Energy Modification

**DOI:** 10.3390/ijms20153812

**Published:** 2019-08-05

**Authors:** A-Rang Lee, Kibeom Nam, Byeong Jun Lee, Seoung-Woo Lee, Su-Min Baek, Jun-Sun Bang, Seong-Kyoon Choi, Sang-Joon Park, Tae-Hwan Kim, Kyu-Shik Jeong, Dong Yun Lee, Jin-Kyu Park

**Affiliations:** 1 Department of Veterinary Pathology, College of Veterinary Medicine, Kyungpook National University, Daegu 41566, Korea; 2 Department of Polymer Science and Engineering, Kyungpook National University, Daegu 41566, Korea; 3 Core Protein Resources Center, Daegu Gyeongbuk Institute of Science and Technology (DGIST), Daegu 42988, Korea; 4 Laboratory of Veterinary Histology, College of Veterinary Medicine, Kyungpook National University, Daegu 41566, Korea; 5 Stem Cell Therapeutic Research Institute, Kyungpook National University, Daegu 41566, Korea

**Keywords:** silica nanoparticles, surface energy modification, NP-based drug delivery

## Abstract

The cellular distribution of silica nanoparticles (NPs) in the liver is not well understood. Targeting specific cells is one of the most important issues in NP-based drug delivery to improve delivery efficacy. In this context, the present study analyzed the relative cellular distribution pattern of silica NPs in the liver, and the effect of surface energy modification on NPs. Hydrophobic NP surface modification enhanced NP delivery to the liver and liver sinusoid fFendothelial cells (LSECs). Conversely, hydrophilic NP surface modification was commensurate with targeting hepatic stellate cells (HSCs) rather than other cell types. There was no notable difference in NP delivery to Kupffer cells or hepatocytes, regardless of hydrophilic or hydrophobic NP surface modification, suggesting that both the targeting of hepatocytes and evasion of phagocytosis by Kupffer cells are not associated with surface energy modification of silica NPs. This study provides useful information to target specific cell types using silica NPs, as well as to understand the relationship between NP surface energy and the NP distribution pattern in the liver, thereby helping to establish strategies for cell targeting using various NPs.

## 1. Introduction

Nanoparticle (NP)-based drug delivery has emerged as a new alternative therapeutic strategy for many diseases [1]. Since the NPs allow the drug to reach whole liver cells such as liver sinusoidal endothelial cells (LSECs), hepatocytes, and Kupffer cells, the cell fate of the NPs is important [2,3]. Drug delivery effects of NPs can vary depending on targeted cell types as well as the locations of targeted cells in the liver. Targeting the right cells can significantly improve the efficiency of NP-based drug delivery and also enhance stability and safety [4]. Conversely, when NPs are mainly engulfed by Kupffer cells, this can lower the level of NPs acting on other cells [2]. Therefore, the selective delivery of NPs to the targeted specific cells is one of the most important issues in NP-based drug delivery. Modification of the NP surface is a universal approach to improving the efficacy of NP targeting to specific cell types [4]. Information on the relative NP uptake quantity per liver cell is essential to ensuring accurate cell targeting.

An NP is a small object with a diameter of 1–500 nm that can be composed of various materials, such as silica, polylactic-co-glycolic acid, and silver [2,5]. NPs are widely applied in medicine and pharmaceutical fields. Particularly, low-toxicity silica NPs are used in many biomedical applications, due to their biocompatibility, low toxicity, and expandable composition [6,7,8]. Precision control of silica particle size, porosity, crystallinity, and shape allows nanostructures to be tuned for a variety of applications [6]. In addition, many possible surface modifications of silica NPs enable the precise control of surface chemistry to regulate drug or chemical loading, and the dispersion, blood circulation, and site-specific targeting of the NPs [9]. The ability to combine these properties makes silica NPs a viable platform for biomedical imaging, analysis, delivery, monitoring, and resection therapies. For instance, silica nanoparticles can be functionalized as contrast agents to enable bio-imaging [10]. NP-based drug delivery systems can also selectively target the tumor area due to improved permeability and retention effects [10]. NPs also have the potential to monitor and treat the disease of interest. Imaging agents are already integrated into nanomaterials and can be easily functionalized to include therapeutic functions [10]. Thus, it is possible to fabricate multimodal silica NPs for therapeutic applications, such as diagnostic imaging components or gene modifying components.

Cancer is the second most common cause of death worldwide, and statistics show that the number of deaths by cancer was 9.6 million (almost 1 in 6 deaths) in 2018 [11]. Existing cancer therapies include radiation, hormones, and chemotherapy to reduce cancer size, and surgical removal of cancer tissue. Among these methods, radiation therapy and surgical removal are commonly used and considered as the most effective treatment for various types of cancer [12]. However, there are still various limits such as the difficulty for early detection of cancer cells and inadequate anticancer drug concentration in target cancer cells, and the inability to monitor treatment response [13]. It is essential to deliver anticancer drugs selectively to the appropriate cells [14]. Thus, several research studies using NP-based drug delivery have emerged over the past years for targeting cancer cells. However, the stability and safety of NPs must be ensured to be applied to humans for therapeutic purposes. In recent years, several studies about NPs being undertaken have completed the toxicity test and have been approved by the Food and Drug Administration (FDA) [2].

Therefore, reducing the toxicity of nanomaterials and improving their efficiency are important in order to develop highly potential therapies in cancer research.

Hepatocellular carcinoma (HCC), a common primary malignant tumor, is the leading cause of cancer-related deaths worldwide [15]. HCC usually occurs in patients with chronic liver disease. Among these, viral hepatitis, non-alcoholic fatty liver disease and alcoholic liver disease are the main causes of HCC [16]. Hepatocytes are not only targets for the treatment of hepatocellular carcinoma, but also have stem cell-like abilities for almost infinite regeneration, which can be a cause of hepatocellular carcinoma [16]. Successful targeting of hepatocytes may enable liver cancer to be treated more efficiently. Therefore, it is important to target hepatocytes specifically for the successful treatment of hepatocellular carcinoma.

Surface charge or hydrophobicity of NPs is one of the most important factors for cell-targeted NP delivery [4,17]. Previous studies have demonstrated that the binding of plasma proteins to NPs is mainly reliant on the hydrophobicity, surface charge, composition, and size of the NP [17,18]. Specifically, the affinity of NPs to albumin, immunoglobulin, fibrinogen, and lipoprotein can affect and determine the NP biodistribution pattern via the bloodstream, as well as the cellular distribution of NPs among various parenchymal and non-parenchymal cells, including immune cells, endothelial cells, and mesenchymal cells [4,19,20]. Moreover, the cellular distribution pattern of the NPs can vary depending on the quantity and properties (e.g., size, surface energy) of the proteins binding to the NPs. Therefore, an evaluation of the cellular distribution pattern of NPs is very important in optimizing cell-targeted NP delivery.

In the liver, the size of the fenestrae in LSECs is estimated to be about 280 nm [21,22]. Thus, NP size should be under 280 nm for NP delivery to hepatocytes [23]. The present study analyzed the relative cellular distribution patterns of silica NPs in the liver, according to NP surface energy modification. We modified the surface polarity of silica NPs to hydrophilic or hydrophobic.

## 2. Results

### 2.1. Silica NP Synthesis, Surface Modification, and Characterization

**Figure 1:**
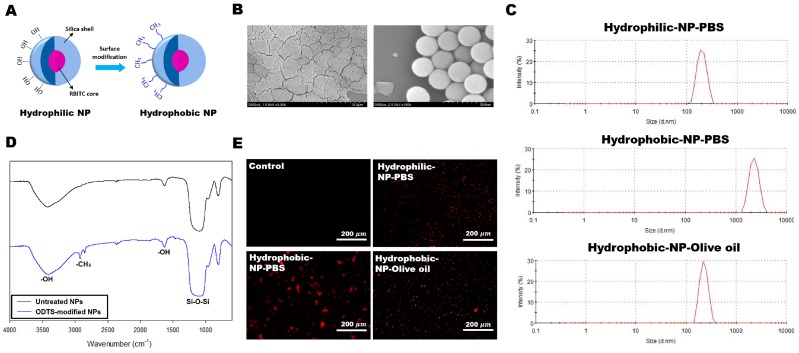
Silica nanoparticle (NP) synthesis, surface modification, and characterization. (**A**) Schematic illustration of rhodamine B isothiocyanate (RBITC)-loaded silica NPs and surface modification with *n*-octadecyltrichlorosilane (ODTS). (**B**) SEM images at (left) low and (right) high magnification of silica NPs. (**C**) Particle size determination by dynamic light scattering (DLS). (**D**) FT-IR spectra of pure silica (black line) and ODTS-modified silica (blue line). (**E**) Fluorescence images of untreated and ODTS-modified silica NPs in different solutions. Dispersion pattern of silica NPs (red) varied based on the characteristics of the NP surface and solvents.

NPs were prepared through the Stöber method, using rhodamine B isothiocyanate (RBITC) as a core structure, to enable NP observation or tracking by fluorescence microscopy (Figure 1A). Since prepared silica NPs have a hydroxyl group on their surfaces, they have a hydrophilic nature. To make hydrophobic silica NPs, aliphatic hydrocarbon groups (i.e., *n*-octadecyltrichlorosilane, ODTS) were attached to the surface of NPs through hydrolysis and condensation reactions (Figure 1A). This surface modification of the silica NPs was conducted to determine the cellular distribution property according to the surface energy of the NPs. Silica NPs have an average uniform diameter of approximately 260 nm (Figure 1B). To identify the exact size of hydrophilic and hydrophobic NPs, we measured particle size in solution environments by dynamic light scattering (DLS) (Figure 1C). When hydrophilic NPs and hydrophobic NPs are dispersed in olive oil and PBS solution, hydrophilic NPs in PBS and hydrophobic NPs in olive oil had similar sizes, whereas hydrophobic NPs dispersed in PBS showed different sizes even though we used the same particles (Figure 1C). This result shows that silica NPs are aggregated by the solvent property and have a different distribution in solution. In the modified silica NPs, C-H_x_ stretching modes at around 2800 and 2900 cm^−1^ are notable compared with those of pure silica, which means that the silane moieties have combined with silica via the silanization reaction (Figure 1D). The stretching vibration of Si-OH was identified near 800 cm^−1^, the Si-O-Si stretching vibration was identified at 1000–1300 cm^−1^, and the peak around 3500 cm^−1^ is the characteristic mode of Si(O-H)_x_ (Figure 1D). RBITC encapsulated in NPs emitted red light at around 590 nm when excited by light generated from the mercury lamp. When the solvent containing the silica NPs was visualized under a fluorescence microscope, only RBITC-containing NPs could be observed (Figure 1E). Interestingly, the dispersion pattern of silica NPs varied, based on the characteristics of the NP surface and the solvents. Untreated silica NPs (hydrophilic-NP-phosphate-buffered-saline (PBS)) dispersed well, without aggregation, due to their hydrophilic property and hydrogen-bonding interactions with the surrounding medium (Figure 1E). However, hydrophobic ODTS-modified silica NPs formed a large aggregated mass when PBS was employed as a solvent (hydrophobic-NP-PBS). It is thought that the ionic PBS buffer solution generated repulsive forces against hydrophobic silica NPs and prevented them from dispersing in the solvent as single particles. When olive oil was adopted as the solvent, ODTS-modified silica NPs (hydrophobic-NP-olive oil) showed much better dispersion than those dispersed in PBS solution. Hence, the polarity of solvents should be considered to disperse silica NPs homogeneously into solutions, depending on the characteristics of the materials.

### 2.2. Hydrophobic Surface Modification of Silica NPs Resulted in Increased NP Delivery to the Liver

**Figure 2:**
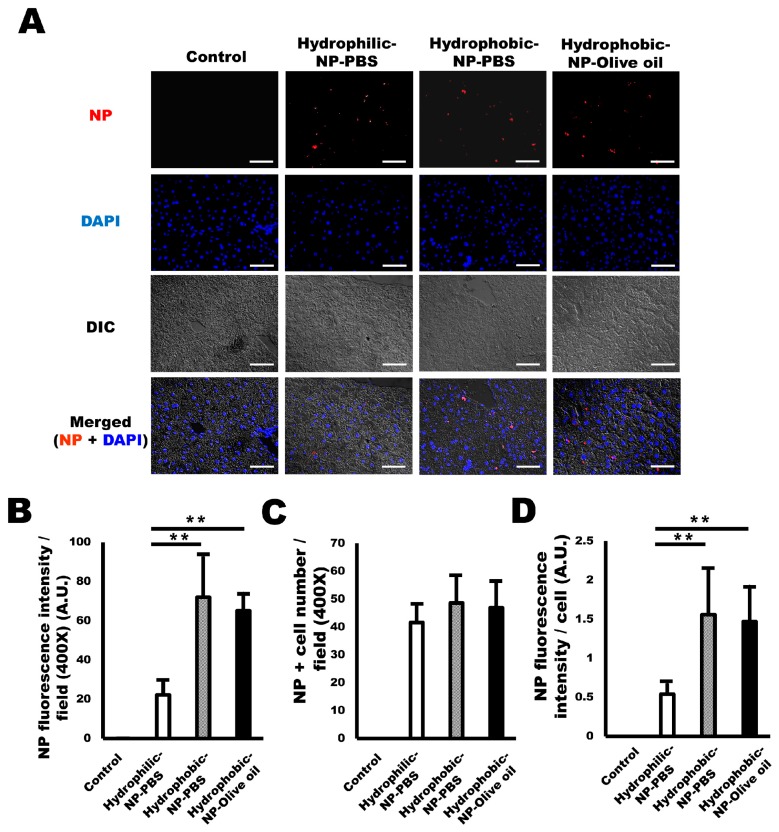
Enhanced NP delivery to the liver by hydrophobic surface modification of silica NPs. (**A**) Representative immunofluorescence micrographs of NPs (red) delivered to the liver. Scale bar = 50 μm. Blue = DAPI. (**B**) NP fluorescence intensity values (arbitrary unit) of NP-treated liver. Hydrophobic NP-treated livers show significantly higher values compared with that of hydrophilic-NP-PBS treated liver. (**C**) The number of NP-positive cells per field based on each surface modification of silica NPs and solvent condition. (**D**) NP fluorescence intensity (arbitrary unit) per cell in NP-treated liver. All data were quantified from 10 fields (400×) per tissue and are shown as mean ± SD. **p < 0.01.

Silica NPs were intraperitoneally injected into mice once, and the mice were sacrificed after 24 h. In the liver sections, NP distribution was detected at 555 nm under a fluorescence microscope. Interestingly, hydrophobic NP-injected mice (hydrophobic-NP-PBS, hydrophobic-NP-olive oil) exhibited a much increased delivery of silica NPs to the liver compared with hydrophilic-NP-PBS (Figure 2A). There was no difference in the NP delivery amount between hydrophobic-NP-PBS treated liver and hydrophobic-NP-olive oil treated liver (Figure 2A). The NP fluorescence intensity value was significantly higher for the hydrophobic NP-treated liver than the hydrophilic-NP-PBS treated liver (Figure 2B). However, the number of NP-positive cells was similar among all groups (Figure 2C), suggesting that the quantity of NPs taken up by each cell might be much higher in hydrophobic NP-treated livers compared with hydrophilic-NP-PBS treated liver. As expected, the NP fluorescence intensity level per cell was significantly higher in hydrophobic-NP-PBS treated liver and hydrophobic-NP-olive oil treated liver relative to that in the hydrophilic-NP-PBS treated liver (Figure 2C). There was no significant difference in the NP fluorescence intensity value between hydrophobic-NP-PBS treated liver and hydrophobic-NP-olive oil treated liver (Figure 2B,D). These results indicate that the delivery of silica NPs to the liver can be improved by preparing silica NPs with hydrophobic surface characteristics.

### 2.3. No Difference Existed in the Ratio of NPs Absorbed by Kupffer Cells among Hydrophilic-NP-PBSTreated Liver, Hydrophobic-NP-PBS Treated Liver, and Hydrophobic-NP-Olive Oil Treated Liver

**Figure 3:**
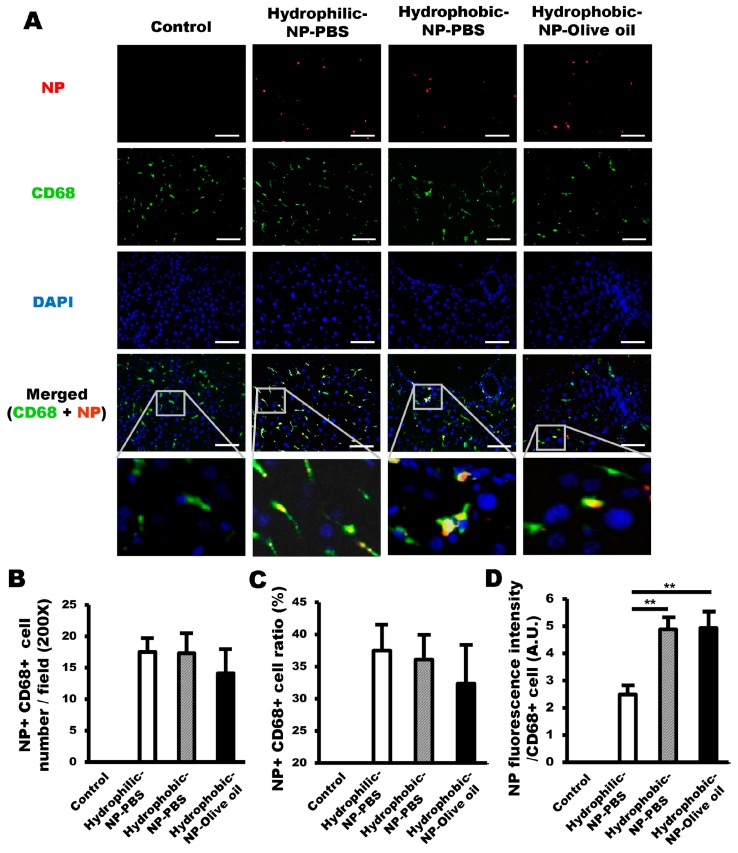
NP uptake by Kupffer cells among hydrophilic-NP-PBS treated liver, hydrophobic-NP-PBS treated liver, and hydrophobic-NP-olive oil treated liver. (**A**) Representative immunofluorescence micrographs of NPs (red) and CD68-positive Kupffer cells (green). Kupffer cells retaining NPs are shown in yellow in the merged images. Scale bar = 100 μm. Blue = DAPI. (**B**) Number of both NP-positive and CD68-positive Kupffer cells per field (200×). (**C**) Ratios of NP-positive Kupffer cells among entire NP-positive cell population. (**D**) Values of NP fluorescence intensity per CD68-positive Kupffer cell in NP-treated liver. All data were quantified from 10 fields (200×) per tissue and are shown as mean ± SD. **p < 0.01.

To determine the cellular distribution of silica NPs, depending on surface characteristics, the NP distribution per each cell type, including Kupffer cells, LSECs, hepatic stellate cells (HSCs), and hepatocytes, was analyzed by immunofluorescence. First, the NP distribution taken up by Kupffer cells was assessed. Accordingly, immunofluorescence with CD68 antibody was used to identify both NP-positive and CD68-positive Kupffer cells (Figure 3A). The NP-positive and CD68-positive Kupffer cells were quantitatively similar among all types of NP-treated livers, without any significant differences (Figure 3A,B). The proportion of NP-positive Kupffer cells among the entire NP-positive liver cell population was constituted by 37 ± 3.9% hydrophilic-NP-PBS, 36 ± 3.7% hydrophobic-NP-PBS, and 32 ± 5.7% hydrophobic-NP-olive oil (Figure 3D). Despite the lack of significant differences in the values among the distinct NP types (Figure 3C), the data suggested that the amount of NPs consumed per Kupffer cell might be higher in the hydrophobic NP-treated liver than in the hydrophilic-NP-PBS treated liver. As expected, the NP fluorescence intensity value per CD68-positive Kupffer cell was significantly higher in hydrophobic-NP-PBS treated liver and hydrophobic-NP-olive oil treated liver in comparison to the hydrophilic-NP-PBS treated liver (Figure 3D). There was no significant difference in the NP fluorescence intensity value between hydrophobic-NP-PBS treated liver and hydrophobic-NP-olive oil treated liver. It inferred that the surface characteristic (hydrophilic or hydrophobic) of silica NPs did not affect their cellular distribution in the liver, although the amount of NPs reaching the liver was greater in the hydrophobic NP-treated liver relative to that of the hydrophilic-NP-PBS treated liver.

### 2.4. NP Delivery to LSECs was Enhanced by Hydrophobic Surface Modification

**Figure 4:**
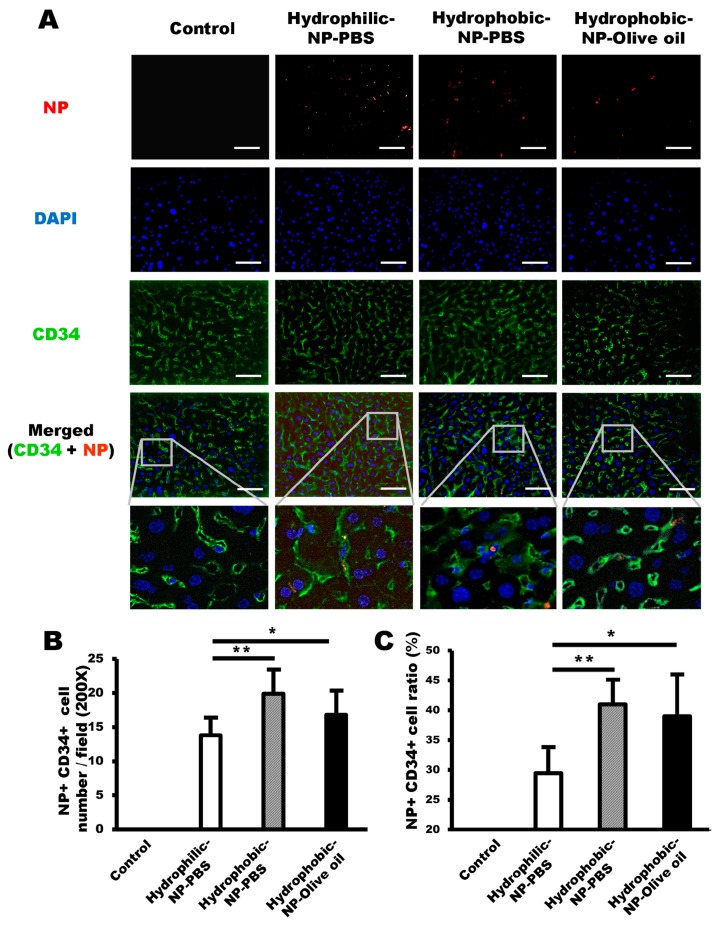
Increased NP delivery to LSECs (liver sinusoidal endothelial cells) by hydrophobic surface modification of silica NPs. (**A**) Representative immunofluorescence micrographs of NPs (red) and CD34-positive LSECs (green). LSECs retaining NPs are shown in yellow in the merged image. Scale bar = 100 μm. Blue = DAPI. (**B**) Number of both NP-positive and CD34-positive LSECs per field (200×). Number of LSECs retaining NPs in hydrophobic-NP-PBS treated liver and hydrophobic-NP-olive oil treated liver was significantly higher compared with that in hydrophilic-NP-PBS treated liver. (**C**) Ratios of NP-positive LSECs among entire NP-positive cell population. All data were quantified from 10 fields (200×) per tissue and are shown as mean ± SD. *p < 0.05. **p < 0.01.

Next, we attempted to analyze NP uptake by LSECs in all types of silica NP-treated liver. Immunofluorescence was performed using CD34 antibody to visualize LSECs taking up the NPs (Figure 4A). There was a significantly higher number of both NP-positive and CD34-positive LSECs in hydrophobic-NP-PBS treated liver and hydrophobic-NP-olive oil treated liver when compared with the hydrophilic-NP-PBS treated liver (Figure 4A,B), possibly implying that silica NPs with a hydrophobic surface might have a higher affinity for LSECs than their hydrophilic counterparts. Surprisingly, the percentage of NP-positive LSECs contributing to the entire NP-positive liver cell population was constituted by 29 ± 4.0% hydrophilic-NP-PBS, 42 ± 4.1 % hydrophobic-NP-PBS, and 39 ± 6.9% hydrophobic-NP-olive oil (Figure 4C). Moreover, the NP-positive LSEC ratio was significantly higher in hydrophobic NP-treated liver than in hydrophilic-NP-PBS treated liver, suggesting that silica NPs with a hydrophobic surface have a greater tendency to be taken up by LSECs than those with a hydrophilic surface.

### 2.5. Hydrophilic Surface Modification of Silica NPs Resulted in Elevated NP Delivery to HSCs

**Figure 5:**
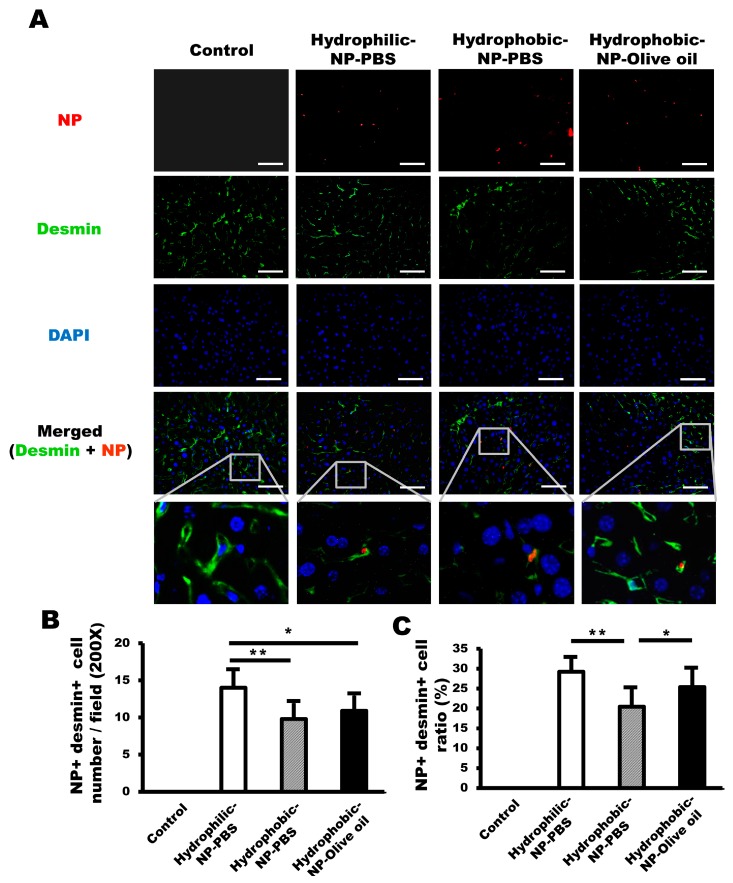
Elevated NP delivery to HSCs (hepatic stellate cells) by hydrophilic surface modification of silica NPs. (**A**) Immunofluorescence image of NPs (red) and desmin-positive HSCs (green). HSCs retaining NPs are shown in yellow in the merged image. Scale bar = 100 μm. Blue = DAPI. (**B**) Number of both NP-positive and desmin-positive HSCs per field (200×). The hydrophilic-NP-PBS treated liver showed the highest number of HSCs retaining NPs. (**C**) Ratios of NP-positive HSCs among entire NP-positive cell population. All data were quantified from 10 fields (200×) per tissue and are shown as mean ± SD. *p < 0.05. **p < 0.01.

Desmin antibody was used to observe NP distribution in HSCs. The overall NP population taken up by HSCs was very low. The number of NP-positive HSCs was significantly higher in the hydrophilic-NP-PBS treated liver (Figure 5A,B), meaning silica NPs with a hydrophilic surface might reach more HSCs compared with hydrophobic silica NPs. As expected, there were significant differences in the percentage of NP-positive HSCs contributing to the entire NP-positive cell population among the three different types of silica NP-treated livers, being 29 ± 3.3% in hydrophilic-NP-PBS treated liver, 20 ± 4.4% in hydrophobic-NP-PBS treated liver, and 25 ± 4.3% in hydrophobic-NP-olive oil treated liver (Figure 5C). The ratio of NP-positive HSCs of hydrophilic-NP-PBS treated liver was significantly higher compared with that of hydrophobic-NP-PBS treated liver and, also, almost significantly (*p* = 0.05) higher compared with that of hydrophobic-NP-olive oil treated liver (Figure 5C). Interestingly, there was also a significant difference in the ratio of NP-positive HSCs between hydrophobic-NP-PBS treated liver and hydrophobic-NP-olive oil treated liver (Figure 5C). Since the ratio of NP-positive HSCs was significantly higher in the hydrophobic-NP-olive oil treated liver compared with that of the hydrophobic-NP-PBS treated liver (Figure 5C), it could be surmised that the differences between the two hydrophobic NP-treated livers could be induced by the different characteristics of the solvent used to dissolve the silica NPs.

### 2.6. Surface Modification of Silica NPs Did Not Affect NP Delivery to Hepatocytes

**Figure 6:**
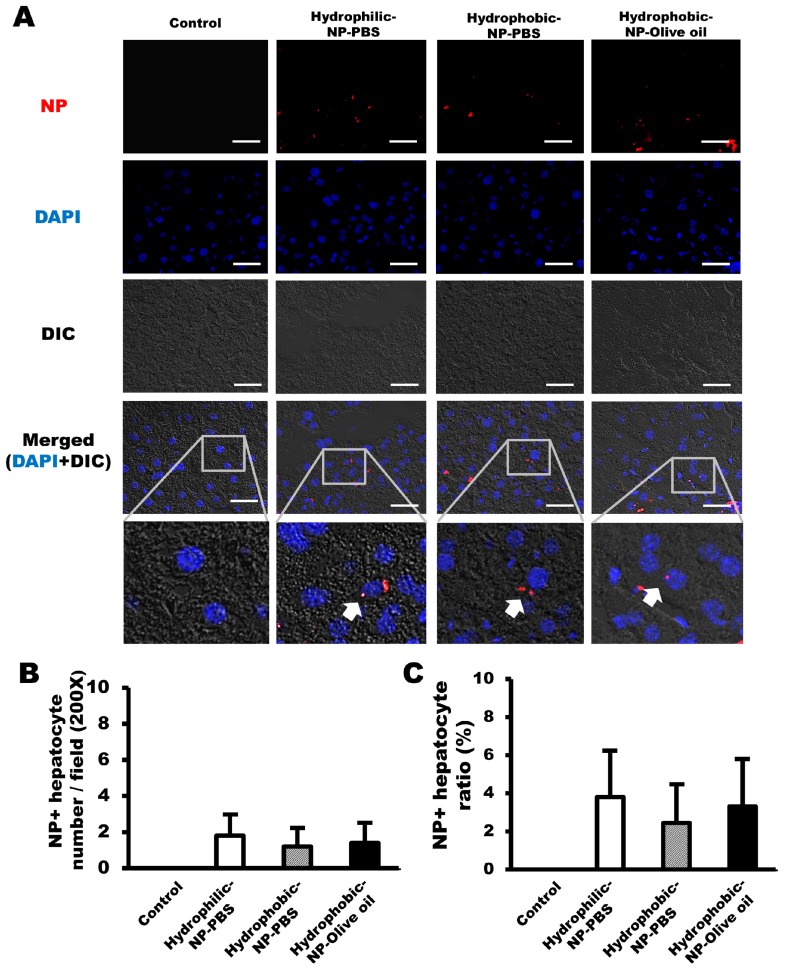
NP delivery to hepatocytes based on surface modification of silica NPs. (**A**) Representative immunofluorescence micrographs of NPs (red) delivered to hepatocytes in the liver. NPs were generally observed in the cytoplasm of hepatocytes around nuclei (blue, DAPI). Blue = DAPI. Gray = Differential Interference Contrast (DIC). (**B**) Number of hepatocytes retaining NPs per field (200×). (**C**) Ratios of NP-positive hepatocytes among entire NP-positive cell population. The ratios were generally less than 4% in all types of NP-treated liver, regardless of NP surface modification and the solvent used to disperse the NPs. All data were quantified from 10 fields (200×) per tissue and are shown as mean ± SD.

Differential interference contrast microscopy and fluorescence imaging (excitation filter, 555 nm) were used to evaluate the NP distribution in hepatocytes. NPs were generally observed around the nuclei of hepatocytes, and the NP population in hepatocytes was very few in all NP-treated livers (Figure 6A). The numbers of NP-positive hepatocytes were almost the same, without any significant difference among all types of NP-treated livers (Figure 6B). The percentage of NP-positive hepatocytes as a proportion of the entire NP-positive cell population was about 4 ± 2.3% in hydrophilic-NP-PBS treated liver, 2 ± 1.9% in hydrophobic-NP-PBS treated liver, and 3 ± 3.3% in hydrophobic-NP-olive oil treated liver, respectively (Figure 6C). Namely, the ratio of NP-positive hepatocytes was less than 4% in all types of NP-treated liver regardless of NP surface modification and the solvent used to disperse NPs.

### 2.7. Hydrophobic-NP-PBS Induced Infiltrations of Inflammatory Cells in the Liver

**Figure 7:**
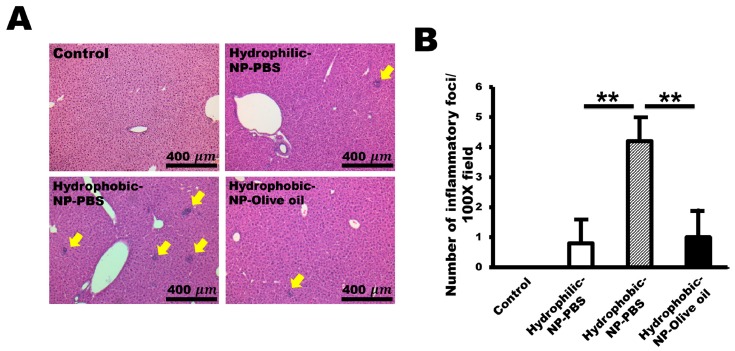
Infiltrations of inflammatory cells in hydrophobic-NP-PBS treated liver. (**A**) Representative histopathologic micrographs of silica NP-injected liver tissue (H&E stain). Hydrophobic-NP-PBS treated liver showed a significantly increased level of inflammatory foci (about 4 or 5 foci per field) compared with those of hydrophilic-NP-PBS treated liver and hydrophobic-NP-olive oil treated liver (arrows). (**B**) Number of inflammatory foci per field (100×) in NP-treated livers. The number of inflammatory foci was counted from 10 fields (100×) per tissue and are shown as mean ± SD. **p < 0.01.

Next, we performed a histopathological analysis of silica NP-injected liver tissue. Silica NPs generally induced a slight infiltration of inflammatory cells into the liver (Figure 7A). To quantify the inflammation, the number of inflammatory foci was counted per 100× field. For the hydrophilic-NP-PBS treated liver and hydrophobic-NP-olive oil treated liver, one or two inflammatory foci were shown per 100× field (Figure 7A). In comparison, hydrophobic-NP-PBS treated liver showed a significantly increased level of inflammatory foci (about 4 or 5 foci per 100× field) (Figure 7A,B). These data suggest that an increased infiltration of inflammatory cells was largely induced by the condensed NP cluster formation of hydrophobic-NP-PBS.

### 2.8. Relative Ratio of NP Uptake by Kupffer Cells, LSECs, HSCs, and Hepatocytes Based on Surface Modification of Silica NPs

Finally, we analyzed the relative ratio of NP uptake based on each cell type and based on the surface modification condition of the silica NPs. In hydrophilic-NP-PBS treated liver, Kupffer cells mainly took up NPs (38%), followed, equally, by HSCs (29%) and LSECs (29%), and then hepatocytes (4%). Interestingly, in hydrophobic-NP-PBS treated liver, LSECs were the main cells taking up NPs (41%), followed by Kupffer cells (36%), HSCs (21%), and hepatocytes (2%). The hydrophobic-NP-olive oil treated liver showed that the hierarchy of NP uptake decreased as follows: LSECs (39%) > Kupffer cells (32%) > HSCs (26%) > hepatocytes (3%). These trends prove that hydrophobic surface modification of NPs may be helpful to target LSECs, whereas hydrophilic surface modification of NPs may enhance their affinity to HSCs. Moreover, there was no notable change in NP uptake ratios between hepatocytes and Kupffer cells by the surface modification of silica NPs, indicating that targeting hepatocytes and evasion of phagocytosis by Kupffer cells are not associated with hydrophilic or hydrophobic surface modification of silica NPs.

## 3. Discussion

The present study determined hepatic cellular distribution of surface-modified silica NPs in mouse liver. Hydrophilic surface-modified NPs dispersed as small-sized particles in PBS (Figure 1B). NPs with hydrophobic surface modification dispersed in olive oil but tended to aggregate in PBS (Figure 1B). When those NPs were intraperitoneally injected, considerably more hydrophobic surface-modified NPs were delivered to the liver compared with hydrophilic surface-modified NPs. Hence, NP delivery to the liver can be improved by hydrophobic surface modification of silica NPs. It is believed that the hydrophobic surface condition may facilitate their absorption by the peritoneal tissues, due to the hydrophobicity of the parietal, visceral peritoneum, mesentery, and omentum covering the abdominal visceral organs [24].

**Figure 8:**
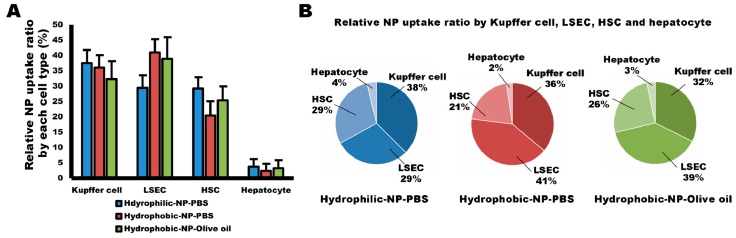
Relative ratio of NP uptake by Kupffer cells, LSECs, HSCs, and hepatocytes based on surface modification of silica NPs. (**A**) Relative NP uptake ratio by Kupffer cells, LSECs, HSCs, and hepatocytes. The ratio values are shown as mean ± SD. (**B**) Hierarchy of NP uptake in all types of NP-treated liver. Kupffer cells mainly took up NPs (38%), followed by HSCs (29%) ≅ LSECs (29%), and hepatocytes (4%) in hydrophilic-NP-PBS treated liver. LSECs were the primary cells taking up NPs (41%), followed by Kupffer cells (36%), HSCs (21%), and hepatocytes (2%) in hydrophobic-NP-PBS treated liver. The hierarchy of NP uptake in hydrophobic-NP-olive oil treated liver was LSECs (39%) > Kupffer cells (32%) > HSCs (26%) > hepatocytes (3%).

In mice, Kupffer cells have been deemed pivotal in removing NPs from the liver [25]. Consistent with this statement, we also observed that NP fluorescence intensity per cell was significantly higher in Kupffer cells than the other types of liver cells, such as LSECs, HSCs, and hepatocytes (Figure S1, Supplementary Materials). Those results can be explained by the cellular location and active phagocytic behavior of Kupffer cells [2]. Interestingly, hydrophilic or hydrophobic surface modification of silica NPs was not associated with NP distribution in Kupffer cells (Figure 3), suggesting that both hydrophobic and hydrophilic surface modification of NPs are not helpful to target and evade Kupffer cells for drug delivery. However, Cheng et al. [26] demonstrated that hydrophobic mesoporous silica NPs were more rapidly removed from liver circulation by Kupffer cells compared with hydrophilic NPs, suggesting hydrophobic NPs rather than hydrophilic NPs were more easily taken up by Kupffer cells. In this study, we synthesized core–shell-type silica NPs and found no significant difference in the relative NP uptake ratio by Kupffer cells among all three types of NP-treated livers (Figure 8). It infers that the affinity of silica NPs for Kupffer cells can be determined by their structure, as well as surface hydrophobicity.

In terms of relative NP uptake ratio by each cell type, LSECs preferentially accumulated the NPs in hydrophobic NP-treated livers (Figure 8B), indicating a hierarchy characterized by the supremacy of LSECs, followed by Kupffer cells, HSCs, and hepatocytes, although the fluorescence intensity per Kupffer cell was much higher when compared with that of the LSECs. Contrary to the hydrophobic NP-treated liver, the hierarchy of relative NP uptake ratio by each cell type in the hydrophilic NP-treated liver was Kupffer cells > HSCs ≅ LSECs > hepatocytes (Figure 8A). Moreover, the ratio of NP-positive LSECs was significantly lower in hydrophilic-NP-PBS treated liver relative to that in hydrophobic NP-treated liver (Figure 4B,C). The fact that hydrophobic silica NPs have a high affinity for LSECs, whereas hydrophilic surface-modified NPs have a low affinity for LSECs means that hydrophobic surface modification of silica NPs can be a pivotal factor in targeting LSECs, specifically in the liver.

LSECs are highly specialized endothelial cells compared with normal continuous endothelial cells in the vessels [27]. LSECs form a permeable barrier, at the interface between the sinusoidal vessels and the space of Disse [28,29]. Therefore, LSECs were regarded as an important barrier to overcoming NP delivery to HSCs or hepatocytes. NP delivery to HSCs and hepatocytes can be impaired by strong endocytosis of LSECs, as well as phagocytosis of Kupffer cells, as evidenced in a previous literature review [30]. This implies a possible convenience of Kupffer cells and LSECs for targeting by NPs, due to their location and strong phagocytic and endocytic activity [31,32]. Based on our results, hydrophobic NP surface modification would be appropriate for targeting LSECs.

Hydrophilic silica NPs seem to be more appropriate to target HSCs rather than LSECs. The number of NP-positive HSCs in the hydrophilic-NP-PBS treated liver was significantly highest among all NP-treated livers (Figure 5B), and so hydrophilic surface-modified NPs might be more likely to reach HSCs when compared with the hydrophobic surface-modified NPs. Besides the surface characteristic of NPs, their size is also a highly important factor affecting NP cellular distribution in the liver [32]. In the liver, NPs should penetrate LSECs to reach HSCs and hepatocytes. NPs mainly pass the LSECs through the fenestrae [32]. Generally, the size of LSEC fenestrae in mice varies between 50 and 280 nm [33], and therefore the NPs we used in the present study could be delivered to HSCs, as well as hepatocytes. However, it is thought that comparatively smaller-sized NPs would be more effective for delivery to HSCs and hepatocytes. Importantly, NPs greater than 280 nm may be highly specific to targeting Kupffer cells. Interestingly, there was a significant difference in the ratio of NP-positive HSCs between hydrophobic-NP-PBS treated liver and hydrophobic-NP-olive oil treated liver. Thus, the NPs’ size depends on the presence or absence of silica NP aggregation. In the absence of NP aggregation, NPs seem to pass the LSEC barrier easily via the fenestrae, due to the smaller size of the NPs relative to the fenestrae diameters.

NP delivery to the HSCs has been one of the most important issues for the treatment of chronic liver fibrosis [34,35,36], a condition induced by the activation of HSCs and their transformation to a myofibroblast-producing collagen-rich extracellular matrix, resulting in severe disruption of the liver architecture. Kupffer cells, LSECs, and a collagenous extracellular matrix have been insurmountable obstacles for NP or drug delivery to HSCs to treat liver fibrosis, despite several studies on this issue [33]. Although some recent research has demonstrated the successful delivery of vitamin A-scaffold lipid or polymer NPs to HSCs by targeting mannose 6-phosphate/insulin-like growth factor II receptor, the collagen type VI receptor, and platelet-derived growth factor β receptor [36,37,38], several issues remain unresolved. For instance, a means of passing through the LSECs or evading phagocytosis of NPs by Kupffer cells is still considered essential for the successful and efficient NP delivery to HSCs in liver fibrosis.

All types of NPs were not delivered properly to the hepatocytes (Figure 6), suggesting that both hydrophobic and hydrophilic surface modifications are not successful strategies for specific targeting of NPs to the hepatocytes. Considerable emphasis has been placed on NP or drug delivery to hepatocytes in treating liver cancers, such as hepatocellular carcinoma [32,39]. To date, several attempts have been made to design NPs for delivery to the hepatocytes, such as galactosylated chitosan NPs [40], galactose derivative-modified NPs [41], and dye-functionalized theranostic NPs [42]. Most of the NPs are actively targeting specific surface receptors of hepatocellular carcinoma cancer cells, for example, the asialoglycoprotein receptor, ganglioside GM1 receptor, epidermal growth factor receptor, and integrin receptor [39]. However, considering the presence of these receptors in non-targeted normal cells, it is still very important to improve the specificity of NP targeting to hepatocellular carcinoma cells and hepatocytes. The effects of hydrophobic or hydrophilic surface modification in NP delivery to hepatocytes have not yet been fully studied. The size of the silica NPs we used was approximately 250–270 nm, which is smaller than the size of LSEC fenestrae, so it is feasible that the NPs can pass through the fenestrae. Therefore, based on the results of this study, we assumed that the surface polarity of silica NPs was not associated with NP delivery to the hepatocytes.

Although it is important to target hepatocytes to treat HCC effectively, various types of nanoparticles with the modified surface charge used in the experiments were not appropriate for targeting hepatocytes. Modification of the surface charge alone did not directly affect the targeting of hepatocytes. In order to deliver nanoparticles to hepatocytes, it is preferable to apply various conditions in combination. In our study, there was a limit that hydrophilic-NP-PBS had less liver reach than hydrophobic surface modification NPs. However, since hydrophilic NPs have overcome the barrier and reached relatively large numbers of HSCs and hepatocytes, it is expected that more NPs will reach the hepatocyte by decreasing the size of the hydrophilic NPs and administering the higher concentration of nanoparticles.

Silica NPs, which have low toxicity, are usually used as a drug delivery system due to the convenience of modifying or functionalizing the NP surfaces [43]. However, it is reported that silica NPs can induce oxidative stress and inflammation [28,44]. Agglomerated silica NPs caused inflammation, in this study, notably associated with the hydrophobic-NP-PBS rather than the hydrophilic-NP-PBS and hydrophobic-NP-olive oil (Figure 7B). Therefore, inflammation does not seem to be associated with the surface modification of NPs. As mentioned above, the size ranges of LSEC fenestrae in mice are between 50 and 280 nm, and the aggregated NPs must be larger than 280 nm. Therefore, it is speculated that the clustered NPs could not pass the LSEC fenestrae properly, due to their relatively substantial size. In sinusoidal vessels, the aggregated NPs can provide more opportunities to stimulate adjacent Kupffer cells to recruit various secondary inflammatory cells around the clumped NPs. Although silica NPs have been known to have low toxicity, aggregation of NPs seems to cause inflammation and toxicity in the liver.

The modification of the NP surface with a high affinity ligand binding to a cell-specific receptor is one of the most frequently used methods to improve cell targeting efficiency [4]. For example, coating of NP with vitamin A has been tried to target HSC in a previous study [45]. However, it was uncertain whether vitamin A-coated NP is beneficial in targeting HSCs, since the distributions of NPs in other types of liver cells were not determined in the previous study. In this study, we investigated the distribution patterns of NPs in liver by modifying surface energy. Interestingly, the size of the NPs along with the surface charge affected cellular distribution in the liver. Thus, the size of the NPs seems to be another important factor in determining specific NP delivery to target cells.

The present study describes, for the first time, the cellular distribution of silica NPs in the liver, based on the hydrophobic and hydrophilic surface modification of the NPs. The distribution of the NPs is influenced by their size and surface characteristics. Modifying the NP surface hydrophobicity is essential to improving NP delivery to the liver Kupffer cells and LSECs, whereas a hydrophilic surface modification is appropriate to deliver NPs to HSCs. Targeting hepatocytes using NPs is more complicated than targeting Kupffer cells, LSECs, or HSCs because of the location and the presence of reticuloendothelial barriers to protect hepatocytes. This study provides information imperative to targeting the specific types of liver cells using silica NPs and understanding the association between NP surface energy and the distribution pattern in the liver. Regardless of NP formulation, it seems the surface polarity of NPs is a crucial factor in determining their cellular distribution pattern in the liver. Thus, our results would apply to other types of NPs, in terms of cellular distribution in the liver, and thereby provides valuable evidence to establish strategies for cell targeting using various NPs.

## 4. Materials and Methods

### 4.1. Chemicals

Tetraethyl orthosilicate (TEOS), NH_4_OH (aqueous solution, 28%), RBITC, and 3-aminopropyltriethoxysilane (APTES) were all purchased from Sigma-Aldrich Co. (St. Louis, MO, USA). Ethanol (EtOH) anhydrous was purchased from Duksan, Inc. (Ansan, Korea).

### 4.2. Synthesis of RBITC-Loaded Core 

RBITC-loaded cores were synthesized by a two-step reaction [46]. First, RBITC was dissolved in EtOH at 5 mg/mL, and then 0.4 mL of RBITC solution was mixed with diluted APTES (8 vol % ethanolic APTES). The isothiocyanate groups in RBITC combined with amines of APTES, forming thiourea groups after reaction at room temperature for 24 h. Afterward, 0.1 mL NH_4_OH was added dropwise to the reacted solution to induce hydrolysis and condensation reactions of ethoxy groups in APTES. After 24 h, RBITC–APTES molecules chemically aggregated together, forming an RBITC-loaded core. Fluorescent cores were separated by centrifugation at 13,000 rpm and re-dispersed in EtOH. The washing steps were conducted repeatedly.

### 4.3. Fabrication, Surface Modification, and Characterization of RBITC-Loaded Core–Shell Silica NPs

RBITC-loaded core–shell silica NPs were synthesized via the Stöber process [47]. First, 0.5 mL of TEOS was dissolved in 5 mL EtOH, with stirring for 10 min (TEOS solution); then, 0.65 mL each NH4OH and RBITC-loaded core–shell silica NPs were mixed with 4 mL EtOH for 10 min (core solution). The core solution was poured into the TEOS solution and stirred for 5 h. As a result, the RBITC-loaded cores were surrounded by TEOS via the hydrolysis reaction between TEOS and APTES. To remove impurities from the RBITC-loaded core–shell silica NPs, a rinsing process (×2) was conducted with water and EtOH. Particles were dispersed after 3 h of sonication. Afterward, 3 wt % ODTS was added to modify the surface of the silica NPs with hydrocarbon groups. Prepared ODTS-attached and untreated silica NPs in EtOH underwent solvent exchange process with PBS and olive oil depending on their uses. Three types of solutions were prepared by dispersing ODTS-attached and untreated silica NPs in PBS and ODTS-attached silica NPs in olive oil. For the solvent exchange, silica NPs dispersed in EtOH were separated from EtOH by centrifugation at 13,000 rpm for 5 min and then EtOH was exchanged with PBS and also EtOH was replaced by olive oil. After an hour of re-dispersion into the exchanged solution, centrifugation and dispersion cycle were conducted two more times. To analyze the functional groups of the silica NPs, the untreated and ODTS-modified silica NPs were examined using a Fourier transform infrared spectrometer (Jasco FT/IR-4100, Easton, MD, USA).

### 4.4. Animals

Thirteen-week-old male C57BL/6 mice weighing 25 g each were housed in individually ventilated cages, maintained at 22–24 °C and 40–50% humidity under 12 h light/dark cycle. Water and normal chow diet were provided ad libitum. For this study, 15 mice were divided into 4 groups (control, hydrophilic-NP-PBS, hydrophobic-NP-PBS, hydrophobic-NP-olive oil) and each group contained 3 or 4 mice. The animals were acclimated under this environment for 7 days before experiments. All animal experiments were performed in accordance with the National Institutes of Health (NIH) guidelines for the care and use of laboratory animals and approved by the Kyungpook National University Institutional Animal Care and Use Committee (IACUC, approval number 2017-0119, 5 May 2017).

### 4.5. NP Preparation and Administration

Three distinct silica NPs were prepared—hydrophilic-NP-PBS, hydrophobic-NP-PBS, and hydrophobic-NP-olive oil. Hydrophilic silica NPs were dispersed in Dulbecco’s PBS, and hydrophobic silica NPs were dispersed in PBS or olive oil. The silica NPs were, respectively, injected intraperitoneally into the mice at a dose of 1.2 mg/25 g. A control group was administered an equivalent volume of Dulbecco’s PBS without NP. All mice were sacrificed for collection of liver tissues at 24 h after a single NP injection.

### 4.6. Immunofluorescence Analysis of Silica NP Distributions in the Liver

For the immunofluorescence observation of NP distribution in the liver, collected liver tissues were fixed in 4% paraformaldehyde at 4 °C for 2 days in the dark and then incubated in 30% sucrose overnight. The tissues sufficiently incubated in sucrose were embedded in OCT compound (Sakura FineTek, Torrance, CA, USA) and rapidly frozen on dry ice. The frozen OCT blocks were cryo-sectioned into 5 μm thick slices. The sections were immunostained with primary antibodies of rat anti-CD68 (Bio-Rad, Hercules, CA, USA), mouse anti-CD34 (Santa Cruz Biotechnology, Santa Cruz, CA, USA), and mouse anti-desmin (Santa Cruz Biotechnology). The sections were subsequently incubated with the secondary antibodies Alexa Fluor® 647 donkey anti-rat IgG and Alexa Fluor® 647 donkey anti-mouse IgG (Abcam, Cambridge, UK). For nuclear staining, ProLong® Gold Antifade Reagent with DAPI (Cell Signaling, Danvers, MA, USA) was used. Silica NP distributions were observed under an Olympus BX53 fluorescence microscope (Olympus, Tokyo, Japan) at 555 nm, and CD68, CD34, and desmin expressions were detected at 647 nm wavelength. The number of NP-positive cells was counted at 200× magnification. For the evaluation of NP intensity per cell, the total NP fluorescence intensity value per field was divided by the number of NP-positive cells or NP-positive specific cell type per field. For example, total NP fluorescence intensity values from CD68-positive cells per field were divided by the number of both NP- and CD68-positive Kupffer cells (CD68+ NP+ cells). Total NP fluorescence intensity was measured under an Olympus BX53 fluorescence microscope using ToupView software (version x86, 3.7.7817, Hangzhou ToupTek Photonics Co., Zhejiang, China).

### 4.7. Histological Analysis of Inflammatory Foci

For histological analysis, isolated liver tissues were fixed in 10% neutral-buffered formalin, routinely processed, and embedded in paraffin wax. The blocks were sectioned to 3 μm thickness. The liver sections were deparaffinized in toluene, rehydrated through graded ethanol solutions, washed in distilled water, and stained with hematoxylin and eosin. Stained tissue slides were observed, and representative images were captured using a Leica microscope (Leica Microsystems, Heerbrugg, Switzerland). The number of inflammatory foci was counted at 100× magnification.

### 4.8. Statistical Analysis

Data were presented as mean ± standard error of the mean. Two-way analysis of variance or the Student’s *t*-test was used to determine the statistical significance among multiple experimental groups, by using GraphPad InStat (GraphPad Software, Inc., San Diego, CA, USA). Statistical significance was set at *p* < 0.05.

## Supplementary Materials

Supplementary materials can be found at https://www.mdpi.com/1422-0067/20/15/3812/s1.

## Abbreviations

APTES	3-aminopropyltriethoxysilane
HSC	hepatic stellate cell
LSEC	liver sinusoid endothelial cell
NP	nanoparticle
ODTS	*n*-octadecyltrichlorosilane
RBITC	rhodamine B isothiocyanate
TEOS	tetraethyl orthosilicate

## References

[1] Liu J., Huang Y., Kumar A., Tan A., Jin S., Mozhi A., Liang X.J. (2014). pH-Sensitive nano-systems for drug delivery in cancer therapy. Biotechnol. Adv..

[2] Park J.K., Utsumi T., Seo Y.E., Deng Y., Satoh A., Saltzman W.M., Iwakiri Y. (2016). Cellular distribution of injected PLGA-nanoparticles in the liver. Nanomed. Nanotechnol. Biol. Med..

[3] Bartneck M., Warzecha K.T., Tacke F. (2014). Therapeutic targeting of liver inflammation and fibrosis by nanomedicine. Hepatobiliary Surg. Nutr..

[4] Kang J.H., Toita R., Murata M. (2016). Liver cell-targeted delivery of therapeutic molecules. Crit. Rev. Biotechnol..

[5] Kim J.W., Kim L.U., Kim C.K. (2007). Size control of silica nanoparticles and their surface treatment for fabrication of dental nanocomposites. Biomacromolecules.

[6] Liberman A., Mendez N., Trogler W.C., Kummel A.C. (2014). Synthesis and surface functionalization of silica nanoparticles for nanomedicine. Surf. Sci. Rep..

[7] Cheng C.J., Tietjen G.T., Saucier-Sawyer J.K., Saltzman W.M. (2015). A holistic approach to targeting disease with polymeric nanoparticles. Nat. Rev. Drug Discov..

[8] Lehman S.E., Morris A.S., Mueller P.S., Salem A.K., Grassian V.H., Larsen S.C. (2016). Silica nanoparticle-generated ROS as a predictor of cellular toxicity: Mechanistic insights and safety by design. Environ. Sci. Nano..

[9] Albanese A., Tang P.S., Chan W.C.W. (2012). The effect of nanoparticle size, shape, and surface chemistry on biological systems. Annu. Rev. Biomed. Eng..

[10] Kramer S.A., Lin W. (2014). Silica-based Nanoparticles for Biomedical Imaging and Drug Delivery Applications. Handb. Nanobiomedical Res..

[11] Siegel R.L., Miller K.D., Jemal A. (2018). Cancer statistic, 2018. CA Cancer J. Clin..

[12] Bayne C.E., Williams S.B., Cooperberg M.R., Gleave M.E., Graefen M., Montorsi F., Novara G., Smaldone M.C., Sooriakumaran P., Wiklund P.N. (2016). Treatment of the Primary Tumor in Metastatic Prostate Cancer: Current Concepts and Future Perspectives. Eur. Urol..

[13] Ahmad K., Rabbani G., Baig M.H., Lim J.H., Khan M.E., Lee E.J., Ashraf G.M., Choi I. (2018). Nanoparticle-based Drugs: A potential Armamentarium of Effective Anti-Cancer Therapies. Curr. Drug Metab..

[14] Egusquiaguirre S.P., Igartua M., Hernández R.M., Pedraz J.L. (2012). Nanoparticle delivery systems for cancer therapy: Advances in clinical and preclinical research. Clin. Transl. Oncol..

[15] Grandhi M.S., Kim A.K., Ronnekleiv-Kelly S.M., Kamel I.R., Ghasebeh M.A., Pawlik T.M. (2016). Hepatocellular carcinoma: From diagnosis to treatment. Surg. Oncol..

[16] Mu X., Español-Suñer R., Mederacke I., Affò S., Manco R., Sempoux C., Lemaigre F.P., Adili A., Yuan D., Weber A. (2015). Hepatocellular carcinoma originates from hepatocytes and not from the progenitor/biliary compartment. J. Clin. Invest..

[17] Aggarwal P., Hall J.B., McLeland C.B., Dobrovolskaia M.A., McNeil S.E. (2009). Nanoparticle interaction with plasma proteins as it relates to particle biodistribution, biocompatibility and therapeutic efficacy. Adv. Drug Deliv. Rev..

[18] Saptarshi S.R., Duschl A., Lopata A.L. (2013). Interaction of nanoparticles with proteins: Relation to bio-reactivity of the nanoparticle. J. Nanobiotechnol..

[19] Gao H., He Q. (2014). The interaction of nanoparticles with plasma proteins and the consequent influence on nanoparticles behavior. Expert Opin. Drug Deliv..

[20] Zhang X., Zhang J., Zhang F., Yu S. (2017). Probing the binding affinity of plasma proteins adsorbed on Au nanoparticles. Nanoscale.

[21] Sarin H. (2010). Physiologic upper limits of pore size of different blood capillary types and another perspective on the dual pore theory of microvascular permeability. J. Angiogenes. Res..

[22] Wisse E., Jacobs F., Topal B., Frederik P., De Geest B. (2008). The size of endothelial fenestrae in human liver sinusoids: Implications for hepatocyte-directed gene transfer. Gene Ther..

[23] Rensen P.C.N., Sliendregt L.A.J.M., Fems M., Kieviet E., van Rossenberg S.M.W., van Leeuwen S.H., van Berkel T.J.C., Biessen E.A.L. (2001). Determination of the upper size limit for uptake and processing of ligands by the asialoglycoprotein receptor on hepatocytes *in vitro* and *in vivo*. J. Biol. Chem..

[24] Gómez-Suárez C., Bruinsma G.M., Rakhorst G., van der Mei H.C., Busscher H.J. (2002). Hydrophobicity of peritoneal tissues in the rat. J. Colloid Interface Sci..

[25] Sadauskas E., Wallin H., Stoltenberg M., Vogel U., Doering P., Larsen A., Danscher G. (2007). Kupffer cells are central in the removal of nanoparticles from the organism. Part. Fibre. Toxicol..

[26] Cheng S.H., Li F.C., Souris J.S., Yang C.S., Tseng F.G., Lee H.S., Chen C.T., Dong C.Y., Lo L.W. (2012). Visualizing dynamics of sub-hepatic distribution of nanoparticles using intravital multiphoton fluorescence microscopy. ACS Nano.

[27] Poisson J., Lemoinne S., Boulanger C., Durand F., Moreau R., Valla D., Rautou P.E. (2017). Liver sinusoidal endothelial cells: Physiology and role in liver diseases. J. Hepatol..

[28] Braet F., Wisse E. (2002). Structural and functional aspects of liver sinusoidal endothelial cell fenestrae: A review. Comp. Hepatol..

[29] DeLeve L.D. (2015). Liver sinusoidal endothelial cells in hepatic fibrosis. Hepatology.

[30] Smedsrød B., Le Couteur D., Ikejima K., Jaeschke H., Kawada N., Naito M., Knolle P., Nagy L., Senoo H., Vidal-Vanaclocha F. (2009). Hepatic sinusoidal cells in health and disease: Update from the 14th International Symposium. Liver Int..

[31] Schroeder A., Levins C.G., Cortez C., Langer R., Anderson D.G. (2010). Lipid-based nanotherapeutics for siRNA delivery. J. Intern. Med..

[32] Poelstra K., Prakash J., Beljaars L. (2012). Drug targeting to the diseased liver. J. Control. Release.

[33] Snoeys J., Lievens J., Wisse E., Jacobs F., Duimel H., Collen D., Frederik P., De Geest B. (2007). Species differences in transgene DNA uptake in hepatocytes after adenoviral transfer correlate with the size of endothelial fenestrae. Gene Ther..

[34] Toriyabe N., Sakurai Y., Kato A., Yamamoto S., Tange K., Nakai Y., Akita H., Harahsima H. (2017). The delivery of small interfering RNA to hepatic stellate cells using a lipid nanoparticle composed of a vitamin A-scaffold lipid-like material. J. Pharm. Sci..

[35] Duong H.T.T., Dong Z., Su L., Boyer C., George J., Davis T.P., Wang J. (2015). The use of nanoparticles to deliver nitric oxide to hepatic stellate cells for treating liver fibrosis and portal hypertension. Small.

[36] Beljaars L., Molema G., Weert B., Bonnema H., Olinga P., Groothuis G.M.M., Meijer D.K.F., Poelstra K. (1999). Albumin modified with mannose 6-phosphate: A potential carrier for selective delivery of antifibrotic drugs to rat and human hepatic stellate cells. Hepatology.

[37] Beljaars L., Molema G., Schuppan D., Geerts A., De Bleser P.J., Weert B., Meijer D.K.F., Poelstra K. (2000). Successful targeting to rat hepatic stellate cells using albumin modified with cyclic peptides that recognize the collagen type VI receptor. J. Biol. Chem..

[38] Beljaars L., Weert B., Geerts A., Meijer D.K.F., Poelstra K. (2003). The preferential homing of a platelet derived growth factor receptor-recognizing macromolecule to fibroblast-like cells in fibrotic tissue. Biochem. Pharmacol..

[39] Varshosaz J., Farzan M. (2015). Nanoparticles for targeted delivery of therapeutics and small interfering RNAs in hepatocellular carcinoma. World J. Gastroenterol..

[40] Zhou N., Zan X., Wang Z., Wu H., Yin D., Liao C., Wan Y. (2013). Galactosylated chitosan–polycaprolactone nanoparticles for hepatocyte-targeted delivery of curcumin. Carbohydr. Polym..

[41] Huang K.W., Lai Y.T., Chern G.J., Huang S.F., Tsai C.L., Sung Y.C., Chiang C.C., Hwang P.B., Ho T.L., Huang R.L. (2018). Galactose derivative-modified nanoparticles for efficient siRNA delivery to hepatocellular carcinoma. Biomacromolecules.

[42] Press A.T., Traeger A., Pietsch C., Mosig A., Wagner M., Clemens M.G., Jbeily N., Koch N., Gottschaldt M., Bézière N. (2014). Cell type-specific delivery of short interfering RNAs by dye-functionalised theranostic nanoparticles. Nat. Commun..

[43] Lee J.A., Kim M.K., Paek H.J., Kim Y.R., Kim M.K., Lee J.K., Jeong J., Choi S.J. (2014). Tissue distribution and excretion kinetics of orally administered silica nanoparticles in rats. Int. J. Nanomed..

[44] Guo C., Xia Y., Niu P., Jiang L., Duan J., Yu Y., Zhou X., Li Y., Sun Z. (2015). Silica nanoparticles induce oxidative stress, inflammation, and endothelial dysfunction in vitro via activation of the MAPK/Nrf2 pathway and nuclear factor-κB signaling. Int. J. Nanomed..

[45] Blomhoff R., Green M.H., Green J.B., Berg T., Norum K.R. (1991). Vitamin A metabolism: New perspectives on absorption, transport, and storage. Physiol Rev..

[46] Ow H., Larson D.R., Srivastava M., Baird B.A., Webb W.W., Wiesner U. (2005). Bright and stable core−shell fluorescent silica nanoparticles. Nano. Lett..

[47] Rossi L.M., Shi L., Quina F.H., Rosenzweig Z. (2005). Stöber synthesis of monodispersed luminescent silica nanoparticles for bioanalytical assays. Langmuir.

