# Supplementary figures and images for: Hepatic Cellular Distribution of Silica Nanoparticles by Surface Energy Modification
Source: Int J Mol Sci. 2019 Aug 5;20(15):3812. doi: 10.3390/ijms20153812 (PMC6696118; doi:10.3390/ijms20153812)

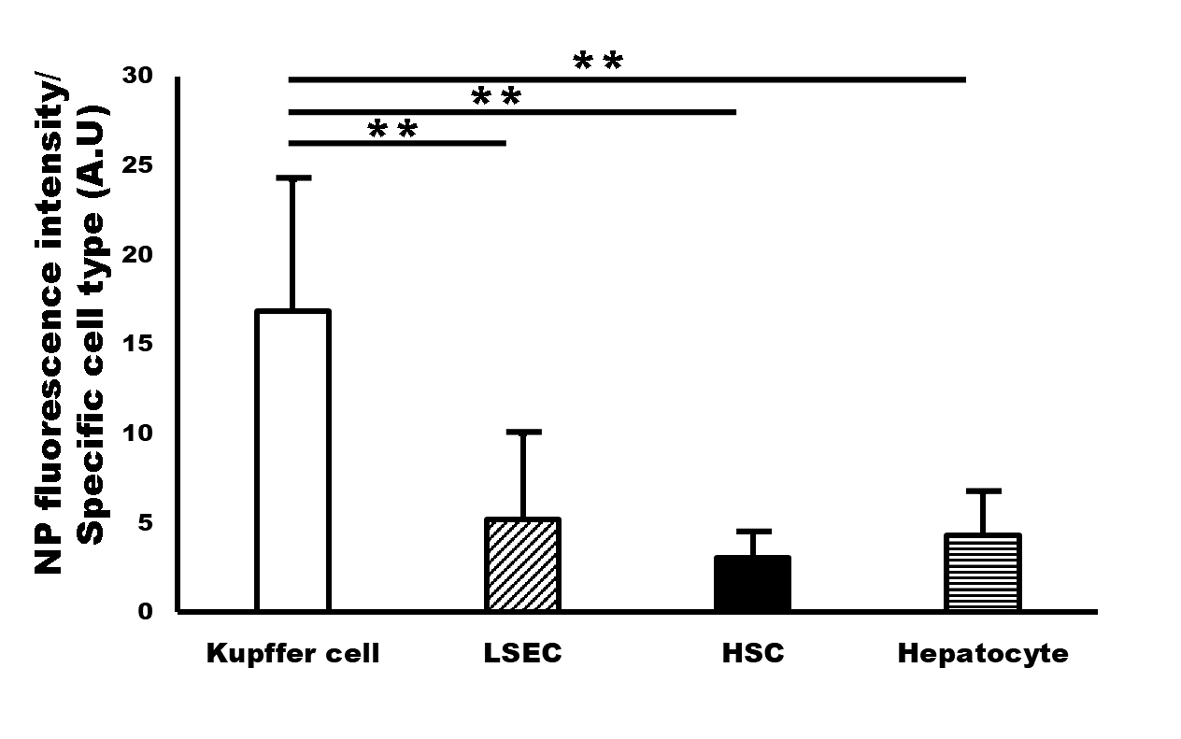

Supplement: Supplementary file 1 [file ijms-20-03812-s001.zip › ijms-538615-SI.gif]
